# Cancer Pathology Turnaround Time at Queen Elizabeth Central Hospital, the Largest Referral Center in Malawi for Oncology Patients

**DOI:** 10.1200/JGO.2015.000257

**Published:** 2017-04-11

**Authors:** Leo P.L. Masamba, Petani E. Mtonga, Linda Kalilani Phiri, Brittany L. Bychkovsky

**Affiliations:** **Leo P.L. Masamba** and **Petani E. Mtonga**, Queen Elizabeth Central Hospital; **Linda Kalilani Phiri**, University of Malawi College of Medicine, Blantyre, Malawi; **Brittany L. Bychkovsky**, Dana-Farber Cancer Institute and Harvard Medical School, Boston, MA.

## Abstract

**Purpose:**

In all settings, a need exists for expedited pathology processing for patients with a suspected cancer diagnosis. In low- and middle-income countries (LMICs) with limited resources, processing pathology samples is particularly challenging, so the measurement of turnaround times (TATs) for pathology results is an important quality metric. We explored the pathology TAT for suspected cancer patients at Queen Elizabeth Central Hospital in Malawi to determine whether a difference exists when patients paid an out-of-pocket fee (paid for [PF] *v* nonpaid for [NPF]) to facilitate sample processing.

**Methods and Population:**

This retrospective descriptive study included all patients with suspected cancer (N = 544) who underwent incisional and excisional biopsy in 2010 at Queen Elizabeth Central Hospital, a teaching hospital in Malawi. Data were abstracted from patient charts and administrative forms to build a database and determine the TAT for PF and NPF samples.

**Results:**

The median TAT for the 544 patients was 71 days (interquartile range [IQR], 31 to 118 days). The median pathology processing time was 31 days (IQR, 15 to 52 days) and was shorter for PF versus NPF samples. The median TAT was 43 days for PF samples (IQR, 27 to 69 days) versus 101 days for NPF samples (IQR, 31 to 118 days), which was significantly different by the Wilcoxon rank sum test (*P* < .01).

**Conclusion:**

The TAT for pathology samples among patients with suspected cancer was longer than reported for other African countries during the study period, was longer than considered acceptable in high-income countries, and differed between PF and NPF samples.

## INTRODUCTION

A pathologic cancer diagnosis is an important step in the care of patients with cancer. Pathology affects all decisions relevant to cancer care: the diagnosis and cancer subtype; the stage; and prognostic information, including whether a patient has curative disease and whether it informs the treatment plan.^1^ In many low- and middle-income countries (LMICs), pathology services often are unavailable, and as a result, patients often are treated without a pathologic diagnosis. Most cancers in sub-Saharan Africa, including Malawi, are diagnosed at an advanced or late stage,^2-5^ and this often is due to both patient and system delays.^6^ When pathology is available in LMICs, system delays often occur in obtaining and processing a biopsy sample, and reporting the results, which consequently delay the start of treatment.^6,7^ Delays in cancer care have a negative impact on outcomes and are a major problem in LMICs with limited resources.^8,9^

In Malawi, delayed cancer diagnoses are a major problem that affect patient outcomes. Like many LMICs, multiple factors in Malawi limit cancer prevention and screening efforts as well as care after diagnosis. The delay from a patient’s first contact with the Malawian health system and initiation of cancer therapy is primarily caused by a delay in processing pathology samples and making the cancer diagnosis. For example, patients at Queen Elizabeth Central Hospital (QECH) in Malawi who undergo a biopsy as part of a cancer evaluation are either hospitalized for a prolonged period or are discharged home without cancer care until the pathology results are reported. An internal audit of patients with breast cancer who presented to QECH in 2012 showed that the average time from onset of symptoms to presentation was 3 months, whereas the average time to confirm a pathologic diagnosis was 5 months. In recognition of this issue, the study pathology turnaround times (TATs) in LMICs is important to identify ways to shorten these TATs and improve outcomes.

No standard definition of pathology TAT exists in the literature. One review defined laboratory TAT as the time from when an investigation was ordered to when the result was published; a diagnostic TAT was defined as the time between when a patient first presents clinically with a symptom or sign that prompts an evaluation of cancer until a pathologic diagnosis was made.^10^ Another study divided TAT into a laboratory (processing of the sample) and a pathology (pathologist interpretation) phase.^11^ Consensus in the literature points to multiple system factors that can prolong TAT, such as delays in submitting a specimen for laboratory processing, inadequately completed requisition forms, time to process and prepare a specimen for microscopic examination, delays in the interpretation and reporting of results, delays in returning the pathology report to the ordering physician, and conveying this information to the patient.^12^

Pathology quality assurance demands that physicians are provided with an accurate, timely, and clinically relevant diagnostic report.^13^ Pathology TAT, therefore, is an excellent metric and partially reflects laboratory quality.^14^ Although we focus on TAT in this article, laboratory quality also encompasses technical and procedural elements as well as overall diagnostic accuracy.^15^ Currently, the American College of Pathologists recommends that pathology TAT be no longer than 2 working days.^16^ This TAT is believed to be a reasonable goal for most routine pathology specimens.

At QECH, pathology samples are sent to the main hospital laboratory after a biopsy is taken. Paid-for (PF) samples are taken directly to the histopathology laboratory after excisional biopsy if the patient pays out-of-pocket fee (US $10). Nonpaid-for (NPF) samples are transferred to the main hospital laboratory where they are labeled in a manner to indicate that the government is responsible for the US $10 processing fee; these samples are then transferred to the histopathology laboratory and not processed until the government pays the US $10 processing fee.

We investigated the TAT for patients who underwent excisional biopsies for a suspected new diagnosis of cancer at QECH in Malawi. The goal was to assess the pathologic TAT defined as the time from which a sample was collected to the time when either the ordering physician or patient received the results. In Malawi, no study to date has investigated the pathology TAT or tried to identify bottlenecks in this process.

## METHODS AND POPULATION

This retrospective study included all patients at QECH in Malawi who underwent an incisional and excisional biopsy between January 1, 2010, and December 31, 2010, for a suspected diagnosis of cancer and had their samples processed by the University of Malawi College of Medicine (COM) histopathology laboratory. We included all inpatients and outpatients who underwent biopsy ordered from either pediatric oncology, adult medical oncology, or surgical departments. We excluded patients who had only a fine needle aspiration as part of their cancer evaluation.

We collected the following data to build our database: patient age, sex, biopsy date, date the sample was transferred to the QECH laboratory, date the sample was received at the COM histopathology laboratory, whether the samples were PF or NPF, date when the pathology report was written, and date when the ordering physician documented receipt of the pathology report. We created a questionnaire to collect these data and had a study team abstract the data from patient charts and internal records.

We defined pathologic TAT as the time from when a biopsy specimen was taken to the time when the ordering physician documented receipt of the results. We broke down TAT into three steps: step 1, the time the biopsy sample was taken to the delivery of the sample to the COM histopathology laboratory; step 2, processing time in the pathology laboratory from arrival of the sample at the COM histopathology laboratory to the time the pathology report was released; and step 3, transmittal time from pathology report release to the time the ordering physician documented receipt of the results. Step 1 included the following two steps: the time for a biopsy sample to be transferred to the QECH main laboratory and the time for the QECH laboratory to transfer the sample to the COM histopathology laboratory. The pathology processing time constitutes the technical processing of the specimen and the time the pathologist takes to read the slides and write the final report. Immunohistochemistry (IHC) staining is not performed routinely on any samples; therefore, the time to perform IHC is not considered in this analysis. All the times in steps 1, 2, and 3 were assessed.

We used Excel software (Microsoft Corporation, Redmond, WA) to build our database. We used Epi Info version 3.4.1.0 (Centers for Disease Control and Prevention, Atlanta, GA) and Stata 10 (StataCorp, College Station, TX) statistical software to calculate means, medians, and interquartile ranges (IQRs). The data were stratified into PF and NPF. The mean and median for steps 1, 2, and 3 and TAT were calculated in each strata. Comparison of medians between PF and NPF samples was performed with the Wilcoxon rank sum nonparametrical test to find the *P* value. The test was two sided, and the statistical significance level was set at α = .05.

## RESULTS

In 2010, 2,985 biopsy samples were processed in the COM histopathology laboratory. Of these, 544 (36.7%) met the inclusion criteria. The median patient age was 37 years (IQR, 24 to 52 years). Fifty percent of the patients were male, 49.6% were female, and 0.4% were unknown sex. Of the 544 samples, 226 (41.5%) were PF, 304 (55.9%) were NPF, and 14 (2.6%) were unknown payment.

For all 544 samples, the median time for a biopsy to reach the QECH main laboratory was 1 day (IQR, 0 to 4 days) and 1 day from the QECH main laboratory to the COM histopathology laboratory (IQR, 0 to 2 days; Table 1). The median pathology processing time for all samples was 31 days (IQR, 15 to 52 days). Median transmittal time was 16 days (IQR, 6 to 42 days). The overall TAT was 71 days (IQR, 31 to 118 days). We also list the means for these data in Table 2.

**Table 1:**
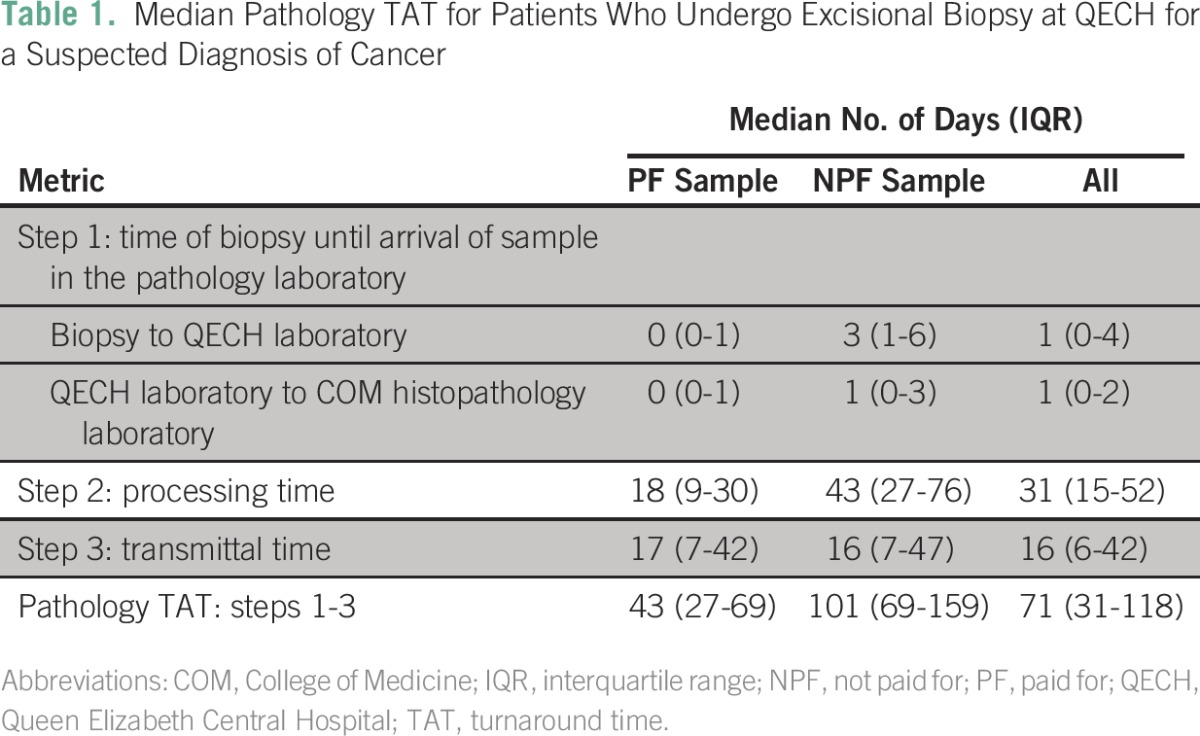
– Median Pathology TAT for Patients Who Undergo Excisional Biopsy at QECH for a Suspected Diagnosis of Cancer

**Table 2:**
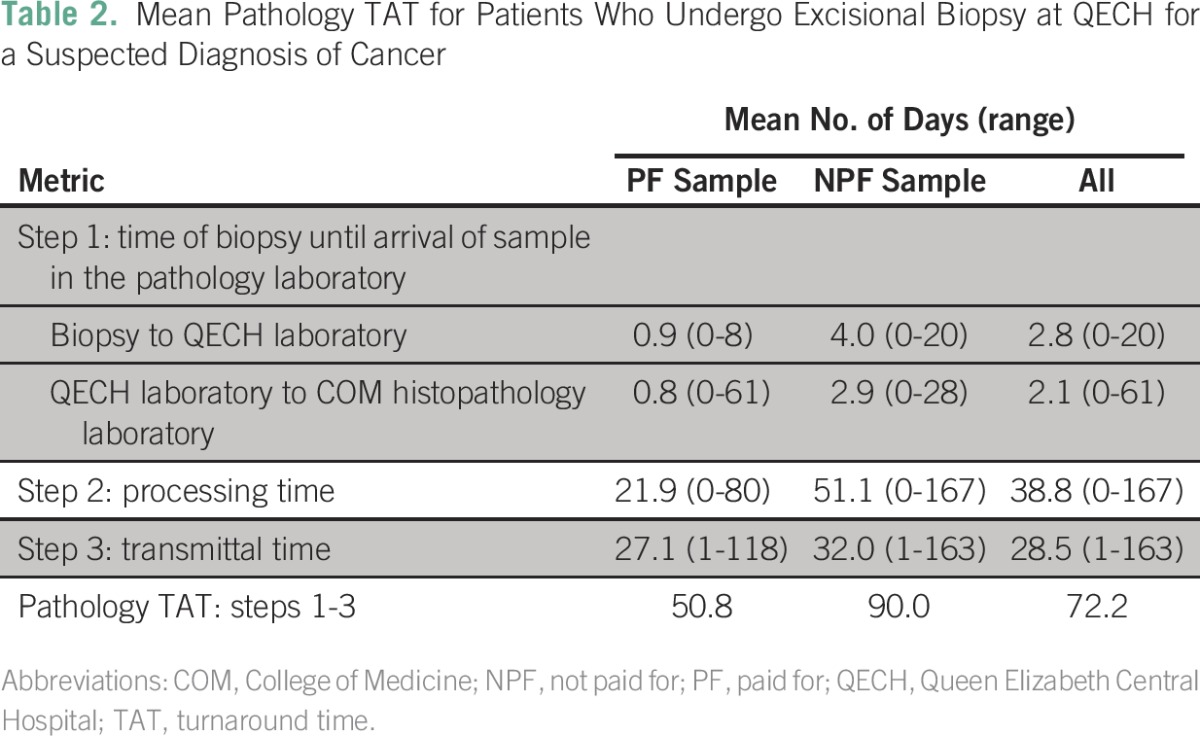
– Mean Pathology TAT for Patients Who Undergo Excisional Biopsy at QECH for a Suspected Diagnosis of Cancer

Table 1 lists the median pathology TAT times for PF and NPF excisional biopsy samples through the various steps at QECH. PF samples reached the COM histopathology laboratory on the same day the biopsy was performed, whereas NPF biopsy samples did not reach the QECH laboratory for a median time of 3 days (IQR, 1 to 6 days). The median time for transferring NPF samples from the QECH laboratory to the COM histopathology laboratory was 1 day (IQR, 0 to 3 days). Once the NPF samples reached the COM histopathology laboratory, the median processing TAT for these samples was 43 days (IQR, 27 to 76 days), whereas the median pathology processing time for PF samples was 18 days (IQR, 9 to 30 days). The median transmittal time for both PF and NPF samples was similar at l7 days and 16 days, respectively.

Overall, the median pathology TAT for the PF samples was 43 days (IQR, 27 to 69 days) and 101 days for the NPF samples (IQR, 69 to 159 days). By the Wilcoxon rank sum test, the difference in TAT between the PF and NPF samples was statistically significant at *P* < .01.

## DISCUSSION

Pathology TAT is an important metric in pathology and clinical medicine because physicians depend on a timely TAT to achieve a diagnosis, develop a treatment plan, and start therapy. In oncology, a timely TAT not only can allow for early initiation of treatment and affect cancer outcomes but also can shorten hospital stays, all of which are cost saving and important in resource-constrained settings.^7,11^ In the current study, the main delay in pathology TAT was due to the processing time (median, 31 days) and transmittal time (median, 16 days).

At QECH, explanations for the long processing time include inadequate staffing, supply shortages, and a high workload. At the time of the study, the COM histopathology laboratory had only two pathologists responsible for teaching undergraduate students, and consequently, only one was available at a time to interpret slides. The COM histopathology laboratory was the only histopathology laboratory in the country, and as a result, it received samples from many clinics and institutions for analysis and was insufficiently staffed and resourced to support the demand. Furthermore, pathology processes that may be automated in high-income countries are not available in LMICs, like Malawi; therefore, the processing of samples is more labor intensive and time consuming.

System issues also played a role in the long processing and transmission times. In the COM histopathology laboratory, no system is in place to help pathologists to identify which samples have been waiting to be processed. The first samples to arrive in the laboratory are not necessarily the first to be processed, so perhaps having a system in place to prioritize the work would help to decrease the TAT. With regard to the transmission time, no established protocol exists for relaying results to the ordering physician, so perhaps standardizing this process would decrease the TAT.

The TAT was significantly less for the PF samples than for the NPF samples (*P* < .01). The processing time for NPF samples was almost twice that of PF samples (43 *v* 18 days). The main reason for a long TAT of the NPF samples was the long processing time. The resources for processing NPF samples rely on state funds, which are usually delayed. However, no statistical difference in transmittal time between NPF and PF samples was found.

Our pathology TAT at QECH and the COM histopathology laboratory is not only much longer than the 2 days recommended by the American College of Pathologists^16^ but also much longer than what other African LMICs have reported. In Muhimbili, Tanzania, the TAT is 5 to 7 days,^17^ but this result cannot be compared directly with our situation because the institution had different resources than QECH and the COM histopathology laboratory, including automated tissue processing and staining, effective management practices, and a computerized system for reporting the pathology results, which clinicians could view online within the wards and clinics.^17^ In 2012, a retrospective study from Butaro in rural Rwanda reported that for pathology TAT, median time from specimen receipt to final reporting was 32 days (IQR, 23 to 44 days; range, 7 to 193 days).^7^ The program in Butaro also differs from QECH because its samples are processed through collaboration with the Dana-Farber Cancer Institute and Brigham and Women’s Hospital in Boston, Massachusetts.^7^ Mpunga et al suggested that the long TAT in Butaro was due to the need to transfer slides internationally for interpretation and special staining, poor Internet connectivity in Rwanda, and lack of a full-time pathologist on site in Rwanda. Although other studies have attributed long pathology TAT to high slide volume/sample numbers or the use of IHC,^18^ these did not contribute to the TAT in the current study because most specimens had three slides prepared for interpretation and IHC was not performed. In the current study, the times between arrival of samples to reporting (technical processing time) and pathologist reporting to transmittal of the results were not possible to determine and constitute the main weakness of the study.

To shorten the pathology TAT at QECH and the COM histopathology laboratory, we recommend the following interventions:

Dedicate personnel to transfer biopsy specimens from the various departments at QECH to the QECH main laboratory and then to the COM histopathology laboratory. Dedicated personnel will help to eliminate the first delay we described, which has a median time of 3 days for NPF samples. Although the median time was 1 day from the QECH main laboratory to the COM histopathology laboratory, some samples stayed ≥ 3 days at QECH before reaching the COM histopathology laboratory.
Develop a system to track samples to process them on a first-in first-out basis, not in batches. This system should include an alert to identify samples that have not been processed and reported in a timely manner (eg, a 15-day threshold).
Increase capacity through more staffing and equipment at the COM histopathology laboratory. There is a recognized need to hire more technicians and pathologists to expedite the processing of pathology samples.
Lobby the state to increase resources to pay for the NPF samples and use a state pathologist with fewer academic commitments to concentrate on interpreting and reporting pathology results.
Purchase and introduce an automated pathology processing system.

An online system for reporting results to clinicians was under development when this study was conducted, and we plan to use this system in a way that will reduce the transmittal time. To do this, we will introduce protocols on how results should be transmitted to patients; for example, the histopathology laboratory sends the results to the hospital, and the hospital staff communicates the results to the patients and/or books a follow-up consultation. Currently, patients directly approach the COM histopathology laboratory for status updates and results.

In conclusion, the pathology TAT measured at a single institution (QECH in Malawi) is suboptimal (median, 71 days [ > 2 months]; range, 1 to 163 days), which negatively affects patient care and raises health care costs. It also increases stress levels for patients and their families who await a diagnosis and treatment plan, especially when their medical providers cannot estimate or predict when a result will be available. We hope this work will prompt future studies in other LMICs in Africa and help to identify creative solutions that will allow patients to receive timely cancer diagnoses. We also hope to bring more awareness to this problem in Malawi and prompt more investment in pathology infrastructure, resources, and training. Since our study was conducted, a collaboration was initiated between the Medical Education Program Initiative and the Malawi government to train staff in pathology and support the development of a pathology laboratory at QECH and a second hospital.^19^ These efforts will likely shorten pathology TAT in Malawi; but these initiatives need to be sustainable because the demand for high-quality pathology will continue to grow. Over the next two decades (from 2015 to 2035), the incidence of cancer in Malawi, similar to most African countries, is predicted to grow by 84%.^20^ In this context, high-quality pathology must be urgently prioritized for cancer care.
